# Tuning Displacement Fields in a Two-Dimensional Topological Insulator Using Nanopatterned Gates

**DOI:** 10.1021/acs.nanolett.4c01518

**Published:** 2024-06-06

**Authors:** Arman Rashidi, Sina Ahadi, Simon Munyan, William J. Mitchell, Susanne Stemmer

**Affiliations:** † Materials Department, University of California, Santa Barbara, California 93106-5050, United States; ‡ Department of Electrical and Computer Engineering, University of California, Santa Barbara, California 93106-5050, United States

**Keywords:** Topological insulator, Rashba spin−orbit coupling, superlattice, Moiré, Landau levels

## Abstract

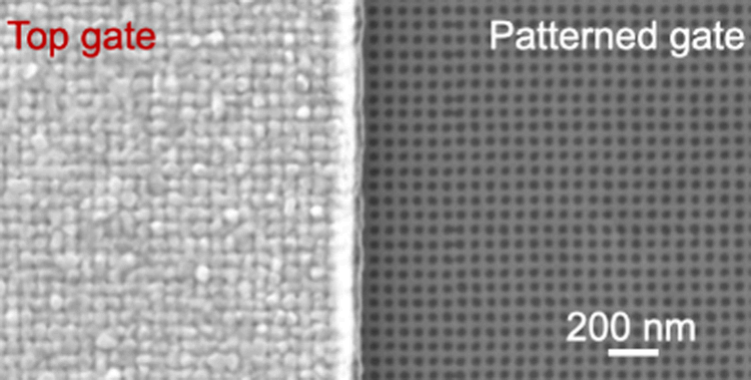

Epitaxial heterostructures with topological insulators enable novel quantum phases and practical device applications. Their topological electronic states are sensitive to the microscopic parameters, including structural inversion asymmetry (SIA), which is an inherent feature of many real heterostructures. Controlling SIA is challenging, because it requires the ability to tune the displacement field across the topological film. Here, using nanopatterned gates, we demonstrate a tunable displacement field in a heterostructure of the two-dimensional topological insulator cadmium arsenide. Transport studies in magnetic fields reveal an extreme sensitivity of the band inversion to SIA. We show that a relatively small displacement field (∼50 mV/nm) converts the crossing of the two zeroth Landau levels in magnetic field to an avoided crossing, signaling a change to trivial band order. This work demonstrates a universal methodology for tuning electronic states in topological thin films.

Realizing quantum spin Hall insulators that host robust helical edge states that can be manipulated in devices is of great interest for novel spintronic and quantum applications.^1−5^ Candidates for quantum spin Hall insulators include atomically thin layers^6−8^ and quantum well structures of two-dimensional topological insulators (2D TIs).^9−14^ While idealized models of 2D TI heterostructures are well established, there are many parameters in practical heterostructures that can result in substantially modified electronic states. One of the most important is structural inversion asymmetry (SIA), which is caused by inequivalent top and bottom interfaces.^14−20^ Strong SIA causes a Rashba-like splitting of the spin-degenerate, massive Dirac bands of the 2D TI, which can destroy the band inversion at *k* = 0 and thus the 2D TI (see Figure 1a). Experimentally, a trivial insulator can be distinguished from the 2D TI by placing the sample in a quantizing magnetic field.^5^ The 2D TI exhibits a crossing of the two lowest (e.g., *N* = 0) Landau levels at a critical magnetic field,^5,9^ due to the inverted band structure. In contrast, in a trivial insulator, these levels show “normal” dispersion in a magnetic field, i.e., they do not cross.^5,21^ Both *N* = 0 Landau level crossings^22^ as well as avoided crossings^23−25^ have been observed in 2D TI thin films. Avoided crossings can also originate from bulk inversion symmetry breaking in noncentrosymmetric crystal structures.^5^

**Figure 1:**
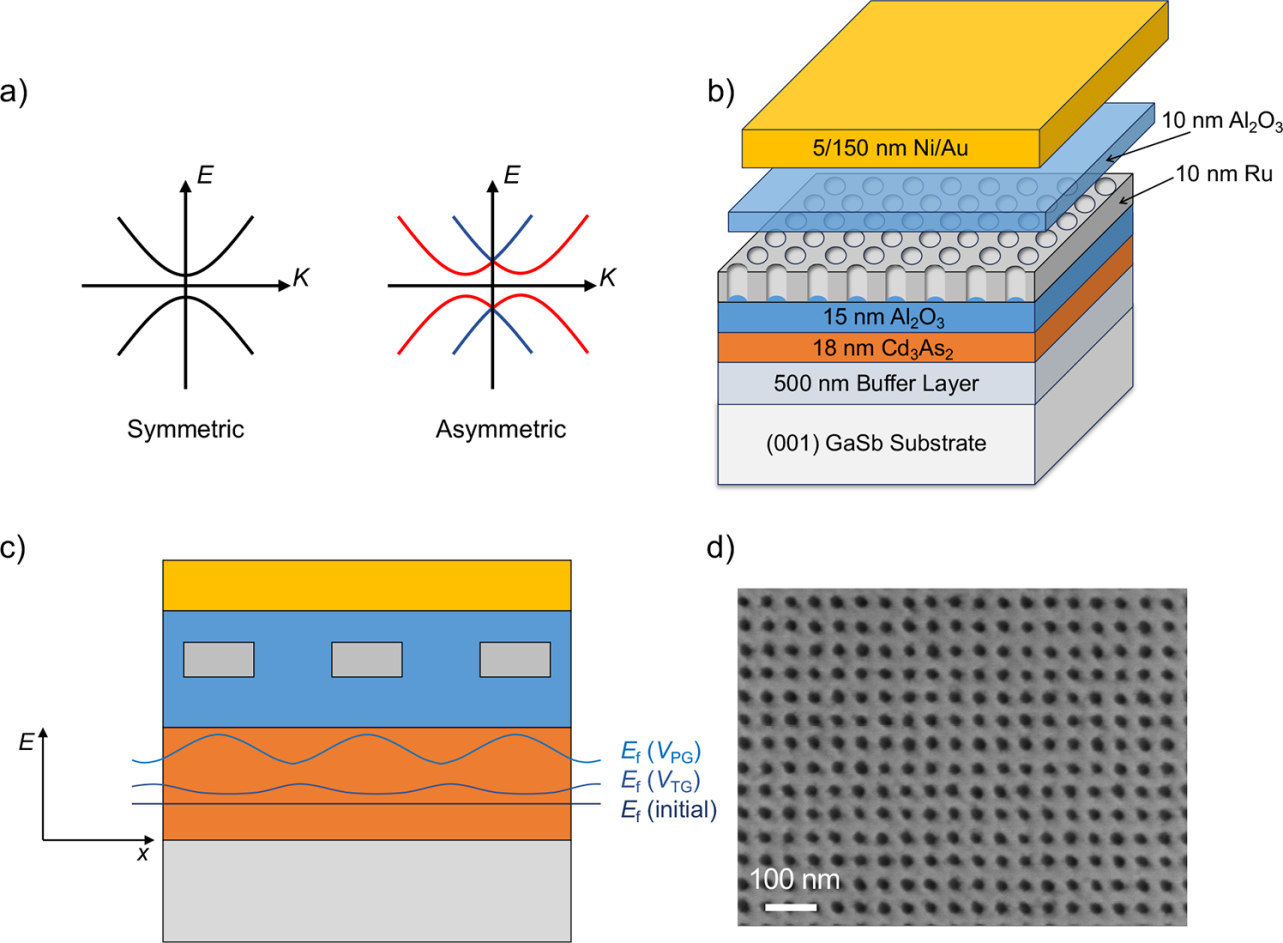
(a) Electronic band structure of a 2D TI without (left) and with (right) SIA, respectively. In the 2D TI all states are doubly degenerate. (b) Schematic of a double-gated device. (c) Fermi level modulation in the Cd_3_As_2_ film due to the gates. The electric field of the top gate extends beneath the patterned gate, controlling the overall Fermi level. (d) A scanning electron microscope image of a patterned Ru gate. The holes are 20 −25 nm in diameter and the pitch is 45 nm.

In principle, the strength of SIA can be controlled by an externally applied electric field. If there is only a single gate, however, the applied gate voltage primarily tunes the carrier density and its main task is to position the Fermi level into the gap of the 2D TI. Thus, to control the Fermi level and displacement field independently, dual gated structures that employ both top and bottom gates are typically used. Unfortunately, effective bottom gating is often difficult to achieve in epitaxial heterostructures, due to various practical constraints, such as lattice matching. For example, high gate leakage through the bottom barrier may become an issue if the barrier has a small band gap or if it contains threading dislocations that serve as leakage paths. Developing an alternative gating architecture that allows for controlling the displacement field in thin films of 2D TIs would be a significant step forward.

Here, we demonstrate a dual-gated heterostructure based on thin films of cadmium arsenide (Cd_3_As_2_), whose film thicknesses are tuned such that they are in the 2D TI state.^22^ Two gates are placed on top of each other, with the first gate being a lithographically pattered gate, while the second gate (“top gate”) modulates the potential through the holes of the patterned gate (see Figure 1b for a schematic of the structure). The top gate is separated from the patterned gate by a dielectric layer. We show that the dual gated structure allows for controlling both carrier density and displacement field. We attribute this result to the small size of the pattern (45 nm pitch and 20–25 nm hole diameter), which allows for the potential from the top gate to extend beneath the patterned gate (see Figure 1c). By reducing the displacement field in the film while keeping the Fermi level constant, we observe a crossing of the *N* = 0 LLs in the magnetoconductance data, consistent with a 2D TI state. In contrast, increasing the displacement field results in an avoided crossing, indicating a transition to trivial band order.

The dual gated devices studied here were fabricated using an 18 nm thick, (001)-oriented Cd_3_As_2_ film grown by molecular beam epitaxy on a (001) Al_0.45_In_0.55_Sb/GaSb buffer/substrate structure. The buffer is lattice-matched to the Cd_3_As_2_ film, which was capped with a thin GaSb layer^26^ (not shown in Figure 1b). Details of the growth and electronic structure have been reported elsewhere.^22,26,27^ Photolithography and ion milling were used to pattern Cd_3_As_2_ mesas into Hall bars. Ohmic contact pads (10/20/200 nm Ti/Pt/Au) were deposited using electron beam evaporation. A 15 nm Al_2_O_3_ gate dielectric was deposited by atomic layer deposition (ALD) at a temperature of 120 °C. Next, a 10 nm ruthenium (Ru) gate metal was deposited by sputtering followed by an 8 nm SiO_2_ mask deposited by ALD. Ruthenium was chosen as the gate metal due to its excellent selective dry etching, while the purpose of the SiO_2_ layer is to mask the Ru metal during the etch.^28^ Electron beam lithography was used to pattern a square lattice of holes with ∼20 nm diameters and a 45 nm pitch in the gated regions. To transfer the pattern to the gate metal, a mixture of CF_4_ and CHF_3_ gases in an inductively coupled plasma was used to etch through the thin SiO_2_ mask followed by an O_2_/Cl_2_ etch to remove Ru in the holes. Finally, Al_2_O_3_ (10 nm) and Ni/Au (5 nm/150 nm) were deposited as the top gate dielectric and top gate metal, respectively. Figure 1d shows a scanning electron microscopy image of a patterned gate. Hall bars without patterned gates were also fabricated on the same sample to serve as a reference.

All measurements were done at 2 K, unless mentioned otherwise, using low-noise lock-in amplifier techniques and low current bias of 0.1–1 nA at a 17.777 Hz frequency. Longitudinal (ρ_*xx*_) and Hall (ρ_*xy*_) resistivities were measured in out-of-plane magnetic fields (*B*) as a function of the patterned gate (*V*_PG_) and the top gate (*V*_TG_) voltages. The resistivities were converted to longitudinal (σ_*xx*_) and Hall (σ_*xy*_) conductivities using tensor inversion:
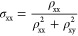
1a
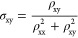
1bFigure 2a shows σ_*xx*_ of an unpatterned Hall bar as a function of the gate voltage (*V*_G_) and magnetic field. The labels indicate the filling factors (ν) determined from the quantum Hall plateaus (see Supplementary Figure S1 for σ_*xy*_ data). The film has a gap at *B* = 0. Two *N* = 0 Landau levels cross at a critical magnetic field (*B*_c_) of 6 T. The crossing is a unique signature for the inverted subband order of the 2D TI (see ref^22^ for a more detailed discussion). At *B* > *B*_c_ the band structure reverts to trivial. The crossing thus corresponds to a topological phase transition.^5,9^ As discussed below and in ref (22), the film is metallic at the crossing point, indicating that it is not an avoided crossing. Thus, SIA is not sufficiently strong to uninvert the band structure of the reference device, despite the asymmetry of the heterostructure.

**Figure 2:**
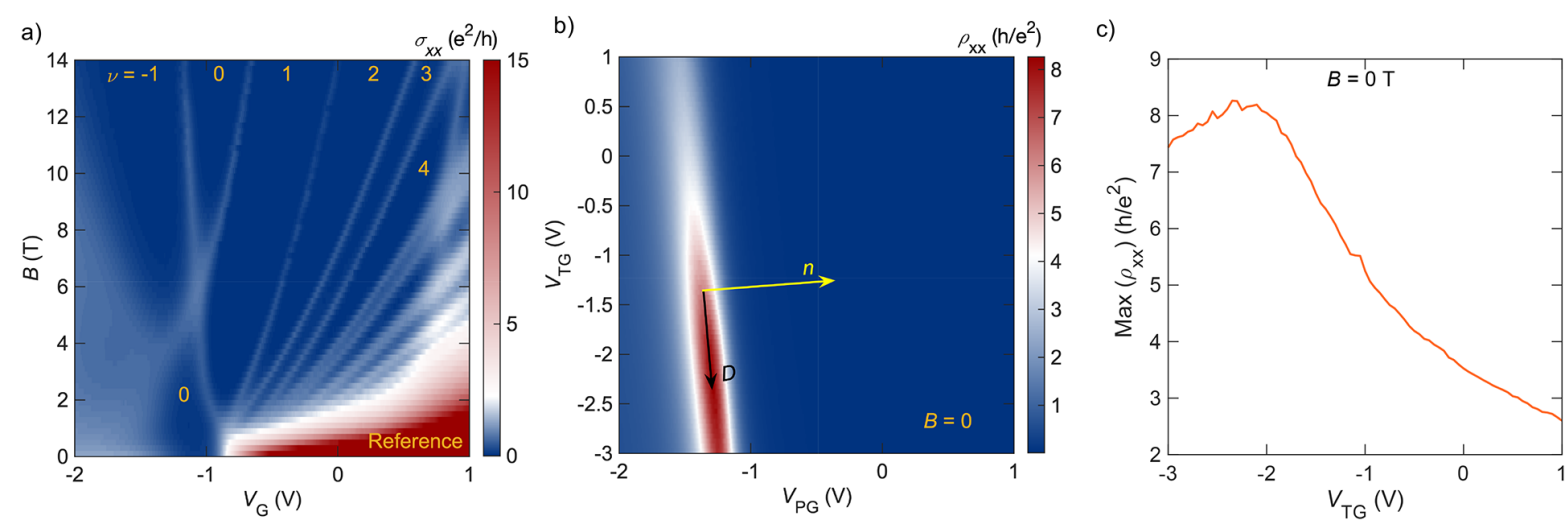
(a) Landau level spectrum of a reference Hall bar device without a patterned gate as a function of gate voltage and magnetic field. The filling factors are obtained from the quantum Hall plateaus (see Supporting Information). (b) Measured longitudinal resistivity of the patterned gate device at *B* = 0 as a function of *V*_TG_ and *V*_PG_. The *D* and *n* axes are drawn as a guide to the eye. Note that *D* = 0 is unknown. (c) Resistivity of the zero-field gap for varying top gate voltages extracted from (b).

Figure 2b shows a ρ_*xx*_ map of a patterned gate device as a function of *V*_PG_ and *V*_TG_ at *B* = 0. Minibands may be expected as a result of the lateral superlattice potential and have been observed in patterned gate devices based on III–V heterostructures (see, e.g., refs^29,30^) and graphene.^31^ Here, no evidence of minibands, which would appear as additional satellite peaks in ρ_*xx*_, can be detected (see Supporting Information and Figure S2 for additional discussion). The resistive region near −1.5 V < *V*_PG_ < −1 V is the gap of the 2D TI. An important feature of the ρ_*xx*_ map is the finite slope of the gap region as a function of *V*_TG_, suggesting that the top gate modulates the carrier density (*n*). This can only be explained by the fact that the top gate modulates the lateral potential beyond the holes, i.e., also beneath the patterned gate. Thus, both patterned and top gates can independently modulate *n* and hence allow for control of the displacement field (*D*) (see Figure 1c). Applying a negative voltage to the top gate is therefore similar to having a positive voltage on a bottom gate in a top-bottom dual-gated structure (and vice versa). To illustrate this point, *n* and *D* axes are shown on the ρ_*xx*_ map in Figure 2b. For instance, *D* can be tuned for a fixed Fermi energy by setting *V*_PG_ and *V*_TG_ in a way that *n* remains constant, i.e., along lines parallel to the *D* axis. From the slope of the *D* axis, we obtain the capacitance ratio of the two gates (*C*_TG_/*C*_PG_), with respect to carrier modulation, to be 0.077. This ratio describes the degree of modulation of *n* by the top gate. The actual capacitance density ratio is estimated to be 0.6 from the thicknesses. We measure an electron density (*n*_0_) of 7.3 × 10^11^ cm^–2^ at *V*_PG_ = 0, which allows us to estimate *C*_PG_ to be 78 nF/cm^2^. The relationship of *n* and *D* with *V*_PG_ and *V*_TG_ is described as follows:
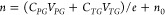
2
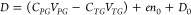
3where *e* is the electron charge and *D*_0_ is the initial displacement field due to SIA, which is unknown. For *n* ≈ 0 (in practice *n* < 1 × 10^10^ cm^–2^), when the Fermi level is in the gap, a total modulation of 54 mV/nm can be achieved in *D* for −3 V < *V*_TG_ < 1 V. The modulation amplitude in *D* is an order of magnitude less than in back-gated van der Waals heterostructures.^32^ It can be improved by increasing *C*_TG_/*C*_PG_, which depends on the thicknesses of the dielectric and gate metal, and the pitch and size of the holes in the patterned gate. The ρ_*xx*_ map (Figure 2b) also shows that the gap at *B* = 0 becomes less resistive when *V*_TG_ is swept toward positive values. This can also be clearly seen in the line cut shown in Figure 2c.

To understand the changes in the electronic structure as a function of *D*, we analyze the Landau level spectrum of the patterned gate device and compare it to that of the reference. Figures 3a-c show σ_*xx*_ maps of the patterned gate device for different top gate voltages. The Landau level spectrum of the patterned gate device differs in two important aspects from that of the reference device. These are the appearance of a resistive feature at high magnetic fields (indicated by the red arrow in Figures 3b and c) and a change in the crossing of the *N* = 0 Landau levels, which we will discuss in detail below. The resistive feature appears at a fixed *V*_PG_ while the rest of the Landau level spectrum shifts with *V*_TG_, as expected due to the dependence of the overall carrier density on *V*_TG_. Given its fixed position, we believe that the resistive feature is not a new feature in the electronic structure of the film (for additional analysis, see Supporting Information Figure S3).

**Figure 3:**
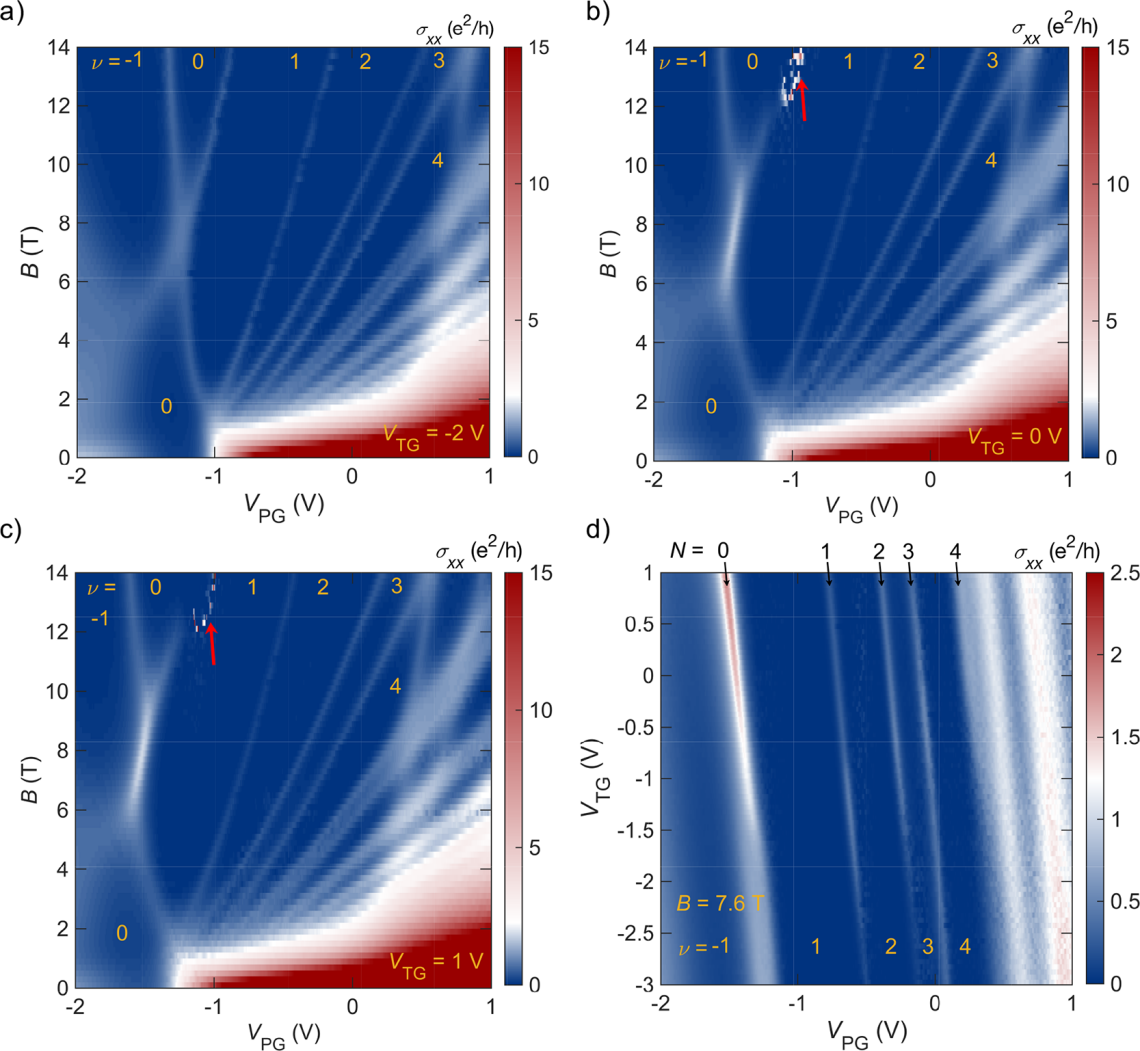
(a - c) Landau level spectrum of a patterned gate device at *V*_TG_ = −2, 0, and 1 V, respectively. The filling factors are obtained from the quantum Hall plateaus (see Supporting Information). (d) Double-gate map of σ_*xx*_ at *B* = *B*_c_ ≈ 7.6 T. Filling factors and the Landau level indices are indicated.

Next, we turn to the topological phase transition associated with the crossing of the two *N* = 0 Landau levels at *B* = *B*_c_. In the patterned gate device, *B*_c_ is 7.6 T, compared to *B*_c_ ≈ 6 T for the reference Hall bar. We attribute the change in *B*_c_ to the stress^33^ imposed on the Cd_3_As_2_ film by the patterned and unpatterned gate metals, respectively. The fact that stress exerted by the gate metal has some influence on the electronic structure, and thus *B*_c_, is apparent also from a comparison of devices with sputtered and thermally evaporated gate metals (see Supplementary Figure S4). The stress is likely lower when the gates are patterned. Importantly, *B*_c_ does not depend strongly on *V*_TG_.

A central finding of this study is the change in the nature of the *N* = 0 Landau level crossing, and therefore band inversion at *k* = 0, as a function of *V*_TG_. Figure 3d shows a σ_*xx*_ map at the crossing (*B* = *B*_c_ ≈ 7.6 T) as a function of *V*_PG_ and *V*_TG_ (ρ_*xx*_ and 1/ρ_*xy*_ maps are shown in Supplementary Figure S5). The width of the crossing increases for *V*_TG_ < −1.5 V, while higher order Landau levels (*N* = 1, 2, 3) do not change notably. For *V*_TG_ > −1.5 V, the crossing becomes more conductive. The two widest Landau levels at positive *V*_PG_ in Figure 3d originate from a different subband (discussed in more detail in ref^22^). They obscure the *N* = 4 Landau level especially when *V*_TG_ < −1.5 V. For better comparison, Figure 4a shows σ_*xx*_ traces of the different Landau levels as a function of *V*_TG_. Higher order (*N* ≠ 0) Landau levels exhibit a small peak at certain *V*_TG_ values (discussed below), while σ_*xx*_ at the *N* = 0 crossing monotonically increases for *V*_TG_ > −1.5 V. Figure 4b shows the resistivity of the crossing as a function of temperature at different *V*_TG_. The resistivity of the *N* = 0 crossing from the reference Hall bar at *B*_c_ ∼ 6 T, which shows metallic behavior (i.e., decrease in resistivity with decreasing temperature), is also included for comparison. In the patterned gate device, for *V*_TG_ ≥ −1 V, the resistivity at the *N* = 0 crossing shows metallic behavior while for *V*_TG_ < −1 V, it becomes insulating. The insulating behavior indicates an avoided crossing for higher displacement fields. The avoided crossing can thus be directly attributed to SIA, in this case induced by the gates. In contrast, for *V*_TG_ ≥ −1 V SIA is minimized and the *N* = 0 Landau levels truly cross. As discussed in the introduction, the change from a crossing to an avoided crossing is indicative of a change of band order from inverted to uninverted (trivial) at the *k* = 0 point. Interestingly, other features, such as *B*_c_ (which depends on the effective mass and g-factor), higher order Landau levels, and the additional subband visible at positive *V*_PG_ are barely affected by the displacement field. This finding shows that the overall electronic structure, unlike the band inversion at *k* = 0, is less sensitive to SIA.

**Figure 4:**
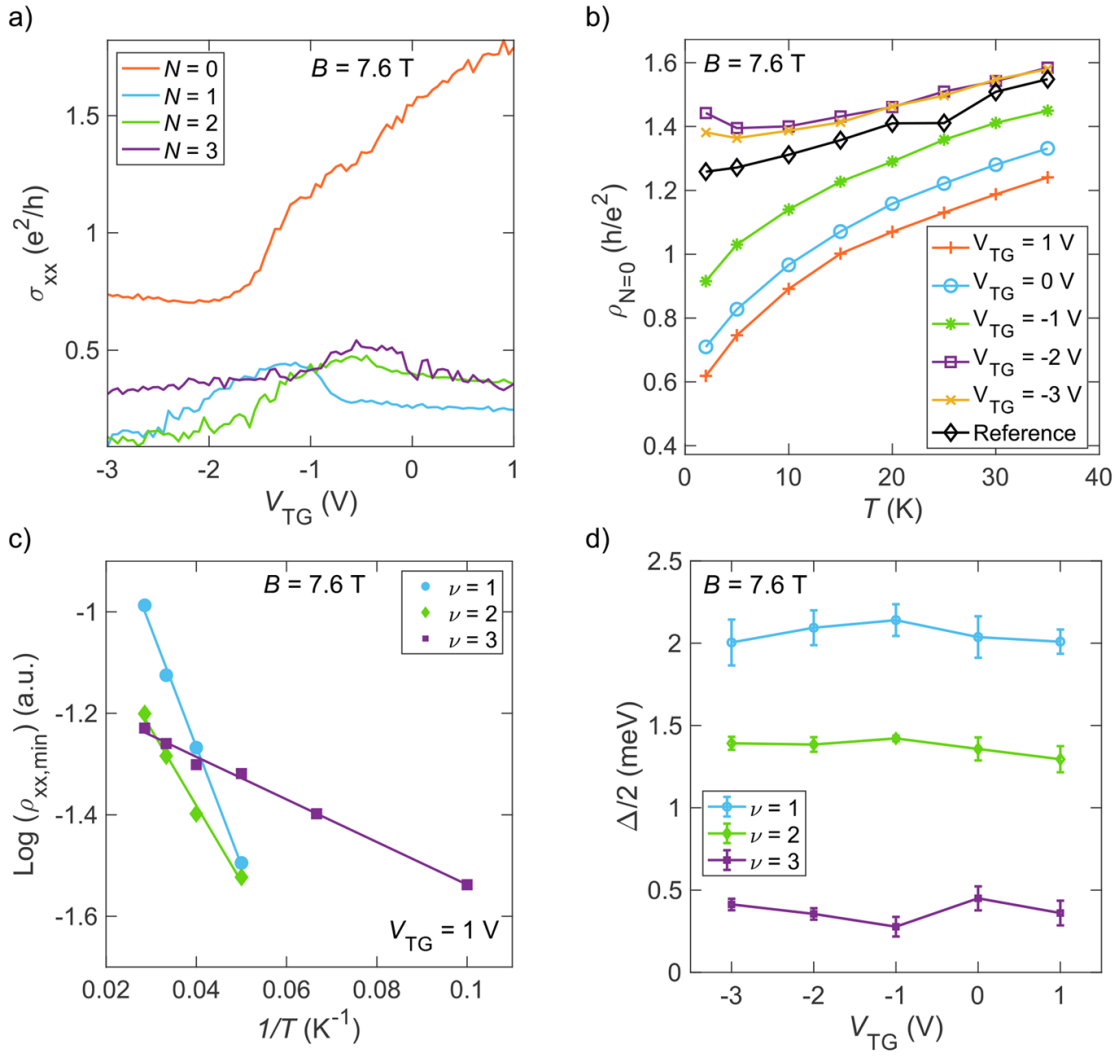
(a) σ_*xx*_ traces of different Landau levels as a function of *V*_TG_, extracted from Figure 3d. (b) Temperature dependence of the *N* = 0 Landau level crossing at different values of *V*_TG_. (c) Arrhenius fits to the minimum of the ρ_*xx*_ at integer filling factors. (d) Half activation energies extracted at different filling factors as a function of the top gate voltage.

As mentioned above, σ_*xx*_ of the higher order Landau levels exhibit maxima at the *V*_TG_ value that is roughly associated with a net zero periodic potential in the film, considering the different dielectric thicknesses of the two gates and the *V*_PG_ at each Landau level. This leads us to consider the lateral disorder created by the top gate.^34^ The influence of the disorder can be probed by studying the broadening of the *N* = 1, 2, 3 Landau levels as a function of *V*_TG_. This broadening is quantified by measuring the half activation energy gap (Δ/2) for quantum Hall plateaus ν = 1, 2, 3 through fitting the minimum of ρ_*xx*_ in the quantum Hall plateaus as a function of temperature using an Arrhenius equation:^35^
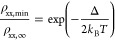
4where *k*_B_ is Boltzmann’s constant, ∞ is a normalization resistivity, and *T* is the temperature. Figure 4c shows the Arrhenius fit for different ν = 1, 2, 3 plateaus at *V*_TG_ = 1 V as an example, while Figure 4d presents the extracted Δ/2 for different values of *V*_TG_. The behavior of the activation energies is mostly independent of *V*_TG_ and does not reflect the much larger changes in the resistivities of the *N* = 1, 2, 3 Landau levels in Figure 4a (the small variations in Δ/2 are on the order of 100 μeV and fall within the uncertainties in the fits). Therefore, we conclude that the disorder potential from the top gate is not large enough to explain the variations in the conductivities observed as a function of *V*_TG_.

In addition to the changes at *B*_c_ (from a crossing to an avoided crossing), the magnitude of the displacement field (SIA) also changes the resistivity of the zero-field (*B* = 0) gap. In particular, reducing SIA when *V*_TG_ is swept toward positive values reduces the resistivity of the gap (Figure 2c). One possible explanation is that when band inversion survives at sufficiently low SIA, the quantum spin Hall edge states contribute to the conductivity when the Fermi level is in the gap. Nevertheless, the resistivity of the gap remains several times higher than the value expected for quantum spin Hall edge modes (*h*/2e^2^). This can be explained with the macroscopic size of our devices, which is much larger than the localization length of such edge modes.^5^

In summary, we have demonstrated that by employing a nanopatterned gate in combination with a top gate, displacement fields and carrier densities can be controlled independently, avoiding the need for a bottom gated architecture. The data provides evidence for the importance of SIA in controlling the topological electronic states of a 2D TI. In particular, we showed that even moderate SIA (displacement fields on the order of 50 mV/nm), which are controlled by the top gate, turns the crossing of *N* = 0 Landau levels of the 2D TI at a critical field into an avoided crossing. This result is indicative of an inverted subband structure becoming uninverted due to SIA. In contrast, other features of the electronic structure are barely affected by SIA. This approach offers a new methodology for tuning band topology and realizing novel quantum phases in TI thin films. For example, in addition to tuning the band order at *k* = 0 discussed here, SIA can control other topological phenomena that rely on inversion symmetry breaking such as topological Hall effects, spin textures and even correlated states.^36,37^

## Data Availability

The data that support the findings of this study are available in the article and its Supporting Information.

## Supporting Information Available

Hall resistivity data from a Hall bar device, discussion of the absence of minibands and the resistive feature at high fields, comparison of sputtered and thermal gates, and Hall resistivity data from the patterned device (PDF)
