# Supplementary material for: Tuning Displacement Fields in a Two-Dimensional Topological Insulator Using Nanopatterned Gates
Source: Nano Lett. 2024 Jun 6;24(24):7366–72. doi: 10.1021/acs.nanolett.4c01518 (PMC11194848; doi:10.1021/acs.nanolett.4c01518)
Supplement: Supplementary file 1 — nl4c01518_si_001.pdf [file nl4c01518_si_001.pdf]

Supplementary Information

Tuning displacement fields in a two-dimensional topological insulator using nano-patterned gates

Arman Rashidi[1], Sina Ahadi[1], Simon Munyan[1], William J. Mitchell[2], and Susanne Stemmer[1*]

[1]Materials Department, University of California, Santa Barbara, CA 93106-5050, USA.

[2]Department of Electrical and Computer Engineering, University of California, Santa Barbara, CA 93106-5050, USA.

Hall resistivity data for reference sample

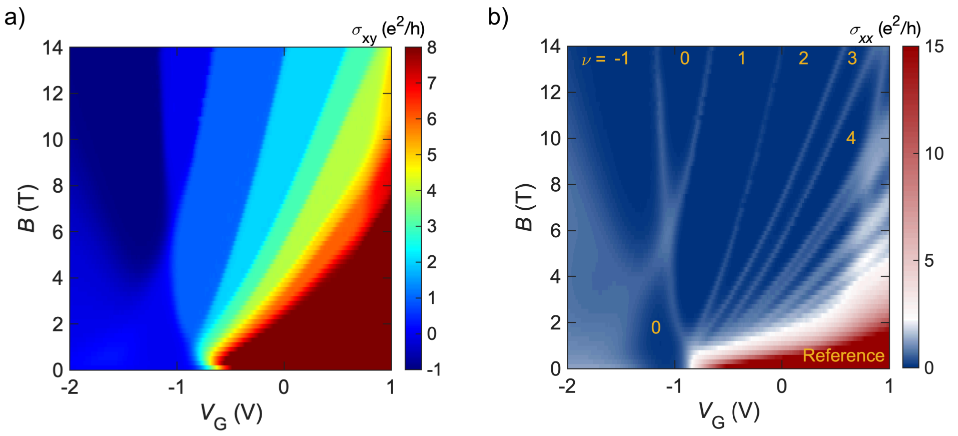

Figure S1. Hall (a) and longitudinal (b) conductivity maps for the reference device. Filling factors are obtained from the Hall plateau values and are indicated in the σ_{xx} map.

Absence of minibands

Minibands are expected to appear as additional satellite peaks in ρ_{xx}. No such peaks are apparent in Fig. 2b in the main text, which shows ρ_{xx} of a patterned gate device as a function of V_{PG} and V_{TG} at a zero magnetic field. The most likely explanation is that such peaks would appear too close to the gap of the 2D TI. In this case, disorder can obscure the minibands [1]. In particular, satellite peaks are expected to appear at carrier densities that are associated with integer fillings, given as multiples of $n^* = 1/A = 5\times10^{10}$ cm^2, where A is the area of the superlattice unit cell. The low value of n^* is within the resistive peak associated with the gap. To visualize this, Figure S2 shows that n^* coincides with the resistive region of the gap of the 2D TI.

Tuning range of the Fermi level

The patterned gate can tune the Fermi level, E_f, from the conduction band across the gap and into the valence band, as evident from the Landau level data. To estimate the range over which E_f is tuned, we calculate E_f at the largest positive patterned gate voltage, V_{PG} = 1 V and V_{TG} = 0 V, which corresponds to a low-field Hall carrier density of n = 1.7×10^{12} cm^{-2}. The estimated value of E_f when it is the conduction band is ~ 20 meV, assuming a conservative Fermi velocity of v_f = 10^5 m/s and $E_f = \hbar v_f \sqrt{2\pi n}$ where $\hbar$ is the reduced Planck's constant. However, the exact strength of the modulation potential (potential difference

between inside and outside the holes) cannot be estimated because it is likely that the patterned gate influences the Fermi level inside the holes as well.

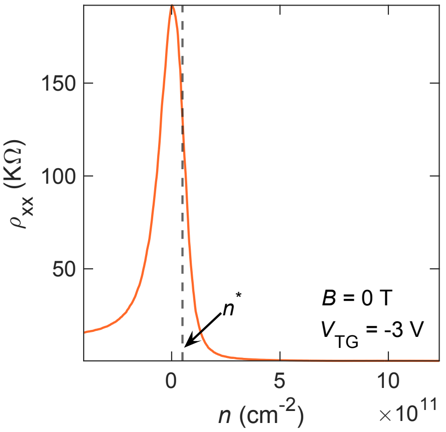

Figure S2. Resistivity of a patterned device *vs.* carrier density for a fixed top gate voltage of -3 V. The n axis is obtained from V_{PG}, using the estimated capacitance densities of the two gates as described in the main text.

Resistive feature at high magnetic fields

Figure S3 shows double-gate maps of σ_{xx} and ρ_{xx} at a magnetic field of 14 T. All Landau levels disperse with V_{TG} as discussed in the main text. The exception is a feature that appears at $V_{TG} > -1.5$ V, which obscures the $N = 0^+$ Landau level (marked by a red dashed line). This feature is a highly resistive state that does not disperse with V_{TG} and appears at a fixed V_{PG}. Figure S3b also shows the artifacts that are a result of heating when the patterned gate tunes the Fermi level into the highly resistive gap at 14 T.

We also consider if the insulating behavior of the anticrossing is influenced by the resistive feature near the $N = 0^+$ Landau level. The effect of the high-field resistive feature should be larger at 11 T compared to 7.6 T because the feature is more resistive at higher fields. Figure S3c shows the σ_{xx} traces at $V_{TG} = 1$ and $V_{TG} = -2$ V at a magnetic field of 11 T. The conductance of the $N = 0$ Landau levels experiences a small increase at $V_{TG} = 1$ from $V_{TG} = -2$ V. This change is much smaller than the change in the conductance of the crossing/anticrossing ($N = 0$) observed in the main text Fig. 4a. Therefore, we conclude that the metallic behavior of the crossing at $V_{TG} > -1$ is not due to the resistive feature moving away from $N = 0$ LLs.

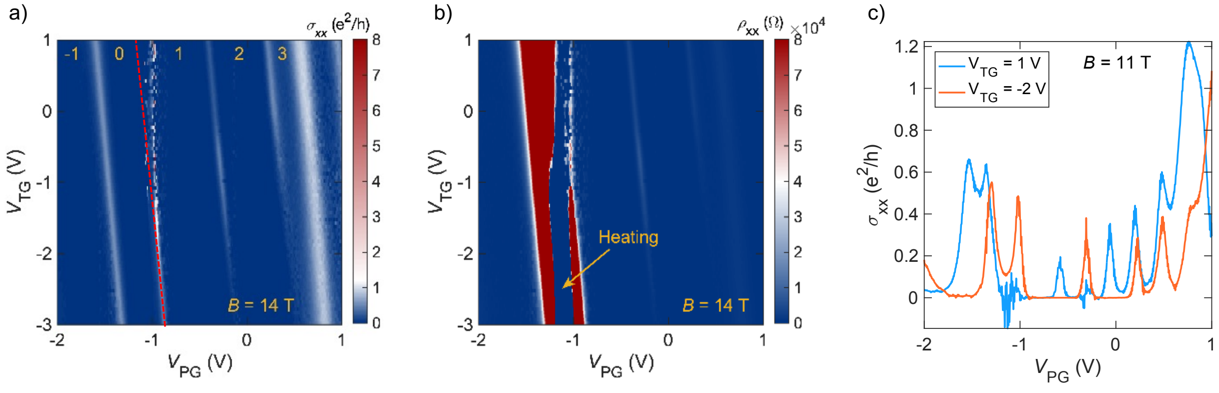

Figure S3. Double-gate maps showing (a) σ_{xx} and (b) ρ_{xx} at a magnetic field of 14 T. (c) σ_{xx} traces for two top gate voltages at 11 T.

Comparison of sputtered and thermally-evaporated gate metals

Figures S4a and S4b show the Landau level maps of an 18 nm Cd_3As_2 film with sputtered Ru and thermally-evaporated Ni/Au (5/150 nm) gate metals, respectively. The critical field at which $N = 0$ Landau levels cross is reduced for the device with a sputtered gate compared with the one with a thermally evaporated gate. The change in the critical field is attributed to the compressive stress that sputtered Ru creates in the film [2].

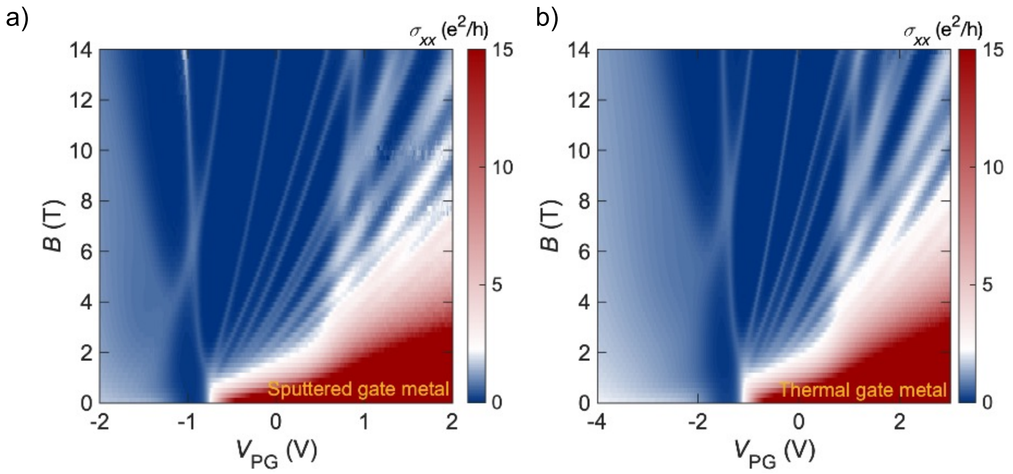

Figure S4. Landau level spectra of an 18 nm Cd_3As_2 film with different gate metals. The gate dielectric thicknesses are different for the two samples.

Hall resistivity data for patterned device at $B = B_c \sim 7.6$ T

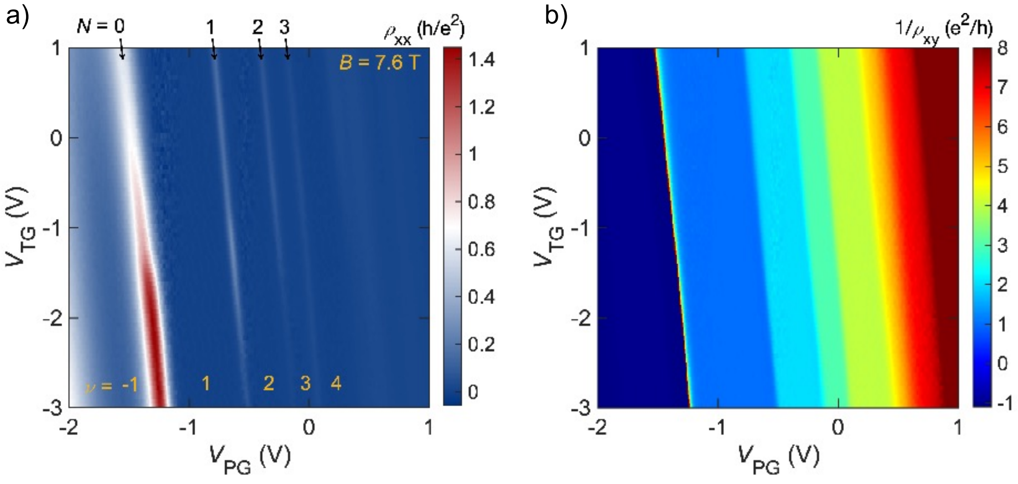

Figure S5. Double-gate map of ρ_{xx} and $1/\rho_{xy}$ at a magnetic field of 7.6 T.

References

[1] C. Forsythe, X. Zhou, K. Watanabe, T. Taniguchi, A. Pasupathy, P. Moon, M. Koshino, P. Kim, and C. R. Dean, *Band structure engineering of 2D materials using patterned dielectric superlattices*, Nat. Nanotech. **13**, 566–571 (2018).

[2] L. B. Freund and S. Sureh, *Thin Film Materials* (Cambridge University Press, Cambridge, 2003).
